# Collagenous gastritis with elevated fecal calprotectin in a pediatric patient

**DOI:** 10.1002/jpr3.12055

**Published:** 2024-02-22

**Authors:** Natalie Rodriguez, Soma Kumar, Jun Mo, Phillipp Hartmann

**Affiliations:** ^1^ Department of Pediatrics University of California San Diego La Jolla California USA; ^2^ Division of Gastroenterology, Hepatology & Nutrition Rady Children's Hospital San Diego San Diego California USA; ^3^ Division of Pathology Rady Children's Hospital San Diego San Diego California USA

**Keywords:** adolescent, case report, chronic abdominal pain, endoscopy, subepithelial collagen bands

## Abstract

Collagenous gastritis is a rare and chronic inflammatory condition of undetermined etiology characterized histologically by thickened subepithelial collagen bands and increased intraepithelial lymphocytes. Here, we present a collagenous gastritis case in a 16‐year‐old female with chronic abdominal pain, persistently elevated fecal calprotectin (507 and 796 mcg/g), and resolved iron deficiency anemia. The patient's history, laboratory tests, endoscopy, and magnetic resonance imaging ruled out common causes of elevated fecal calprotectin, including *Helicobacter pylori* and gastrointestinal infections, medications, celiac disease, and inflammatory bowel disease, as well as less common causes such as collagenous *colitis*. Esophagogastroduodenoscopy revealed significant antral nodularity. Gastric biopsies showed thickened subepithelial collagen band and surface epithelium damage with increased intraepithelial lymphocytes. The ileocolonoscopy was normal. This is among the first reported cases of collagenous gastritis with elevated fecal calprotectin levels that could solely be attributed to this condition.

**Abbreviations**
EGD: esophagogastroduodenoscopy
IBD: inflammatory bowel disease
MRE: magnetic resonance enterography
NSAIDs: nonsteroid anti‐inflammatory drugs

## 1 INTRODUCTION

Collagenous gastritis is a rare inflammatory condition with a prevalence of 13 cases per 100,000 esophagogastroduodenoscopies (EGD).^1^ Diagnosis is based on identifying a subepithelial thickened collagen band ≥10 µm and inflammatory infiltration of gastric lamina propria. In adolescents, the disease often affects only the gastric mucosa and presents with abdominal pain and iron‐deficiency anemia.^2^

Calprotectin is a leukocyte intracellular protein abundant in neutrophils and thus serves as a marker of inflammation. Fecal calprotectin is commonly used to assess inflammation in inflammatory bowel disease (IBD).^3^ However, other causes of elevated fecal calprotectin include infection, malignancy, rheumatological disease,^4^ non‐IBD gastrointestinal disorders such as microscopic colitis including lymphocytic and collagenous subtypes, medications such as nonsteroid anti‐inflammatory drugs (NSAIDs), and age with higher levels seen in older people and neonates.^5^, ^6^, ^7^, ^8^

Three cases of collagenous gastritis with elevated fecal calprotectin have previously been described. In each case, diarrhea or hematochezia was present; in two cases, collagenous *colitis* coexisted.^9^, ^10^, ^11^ Herein, we report on a case of collagenous gastritis with elevated fecal calprotectin without collagenous *colitis* or significant lower gastrointestinal symptoms.

## 2 CASE REPORT

A 16‐year‐old non‐Hispanic white female, born in the United States with no recent travel, BMI‐for‐age at the 94th percentile, history of multiple years of episodic abdominal pain, presented with 5 months of worsening abdominal pain and elevated fecal calprotectin (507 mcg/g). Medical history revealed iron‐deficiency anemia for 4.5 years, starting at 11 years old (Hgb 7.8 g/dL), before onset of menarche at age 15. Anemia largely improved with 1 year of 130 mg/day elemental iron in Ferrous Sulfate pill form despite developing menorrhagia at 15 years old. At presentation, the patient was still on elemental iron and had a Nexplanon in place for menorrhagia for >1 year. Her hemoglobin (14.5 g/dL), mean corpuscular volume (90 fL), and iron levels were normal. Infectious stool studies, including *Helicobacter pylori* antigen, were negative. Tissue transglutaminase IgA (tTg‐IgA) was <2 U/mL, but the IgA level was low (Table 1). Her medication history was negative for NSAIDs, acetylsalicylic acid, or proton pump inhibitors.

**Table 1:** Course of results to presentation.

	Reference range	−4.5 years	−3 years		−1.8 years	−1.5 years	−0.5 years	−0.3 years	0	
Blood tests										
Hemoglobin	10.8–15.0 g/dL	7.8^a^, ^b^	9.4^a^		9.3^c^	10.5^d^	13.8^e^	14.5		n/a
MCV	77–91 fL	n/a			72	77	87	90		n/a
Reticulocyte percent	0.90%–1.49%	n/a			1.53	1.53	1.15	n/a		
Iron	28–173 mcg/dL	n/a			12	15	58	n/a		
Transferrin	202–364 mg/dL	n/a			309	n/a				
TIBC	250–450 mcg/dL	n/a			386	n/a				
Iron saturation	20%–50%	n/a			3	4	17	n/a		
Ferritin	8.0–252.0 ng/mL	n/a			2.8	4.7	17.6	n/a		
WBC	4.2–9.4 K/mcL	n/a						7.8	n/a	
Platelet count	194–345 K/mcL	n/a						257	n/a	
Allergen egg, whole	<0.10 kU/L	n/a						<0.10	n/a	
Allergen soybean	<0.10 kU/L	n/a						<0.10	n/a	
Allergen wheat	<0.10 kU/L	n/a						<0.10	n/a	
Allergen almond	<0.10 kU/L	n/a						<0.10	n/a	
Allergen codfish	<0.10 kU/L	n/a						<0.10	n/a	
Allergen crab	<0.10 kU/L	n/a						<0.10	n/a	
Allergen milk	<0.10 kU/L	n/a						0.12^f^	n/a	
Allergen peanut	<0.10 kU/L	n/a						<0.10	n/a	
Allergen shrimp	<0.10 kU/L	n/a						<0.10	n/a	
Allergen tuna	<0.10 kU/L	n/a						<0.10	n/a	
Allergen walnut	<0.10 kU/L	n/a						<0.10	n/a	
IgE	<114 kU/L	n/a						85	n/a	
TTG IgA	0–3 U/mL	n/a						<2	<2	
IgA	65–421 mg/dL	n/a						38	39	
TSH	0.47–3.41 mcU/mL	n/a						1.78	n/a	
T4, free	0.70–1.48 ng/dL	n/a						1.09	n/a	
Albumin	3.5–4.9 g/dL	n/a						4.1	n/a	
Stool tests										
Ova and parasite		n/a						⊖	n/a	
Giardia antigen		n/a						⊖	n/a	
Salmonella DNA		n/a						⊖	n/a	
Campylobacter DNA		n/a						⊖	n/a	
Shigella DNA		n/a						⊖	n/a	
Shiga toxin DNA		n/a						⊖	n/a	
*H. pylori* Ag		n/a						⊖	⊖	
Fecal calprotectin	≤49 mcg/g	n/a						507	796	
Guaiac stool		⊖		n/a						

Abbreviations: 0, time of presentation; ⊖, negative; Ag, antigen; *H. pylori, Helicobacter pylori*; IgA, Immunoglobulin A; MCV, mean corpuscular volume; n/a, not available (not tested); T4, thyroxine; TIBC, transferrin iron binding capacity; TSH, Thyroid stimulating hormone; TTG, tissue transglutaminase; WBC, white blood cells.

a Premenarche.

b Daily multivitamins with Iron started.

c Transitioned from multivitamin with iron to 130 g/day elemental iron in ferrous sulfate pill form. Now, 7 months from menarche, but no menorrhagia.

d Onset of menorrhagia. Continuing 130 g/day elemental iron.

e On Nexplanon for past 8 months (in addition to continuation of 130 g/day elemental iron).

f Interpretation scale: 0.10–0.34 kU/L – Class 0/1 allergen = low level of allergen‐specific immunoglobulin E (no allergen skin prick test done).

Our patient endorsed daily moderate and intermittently severe sharp lower abdominal pain in the mornings or an hour after meals, occasionally associated with mild nausea. She had some improvement by limiting lactose in her diet and using Lactaid. The physical exam was unremarkable. Fecal calprotectin was 796 mcg/g when repeated. Fecal *H. pylori* antigen and tTg‐IgA with IgA level were equivalent results to prior (Table 1).

**Figure 1:**
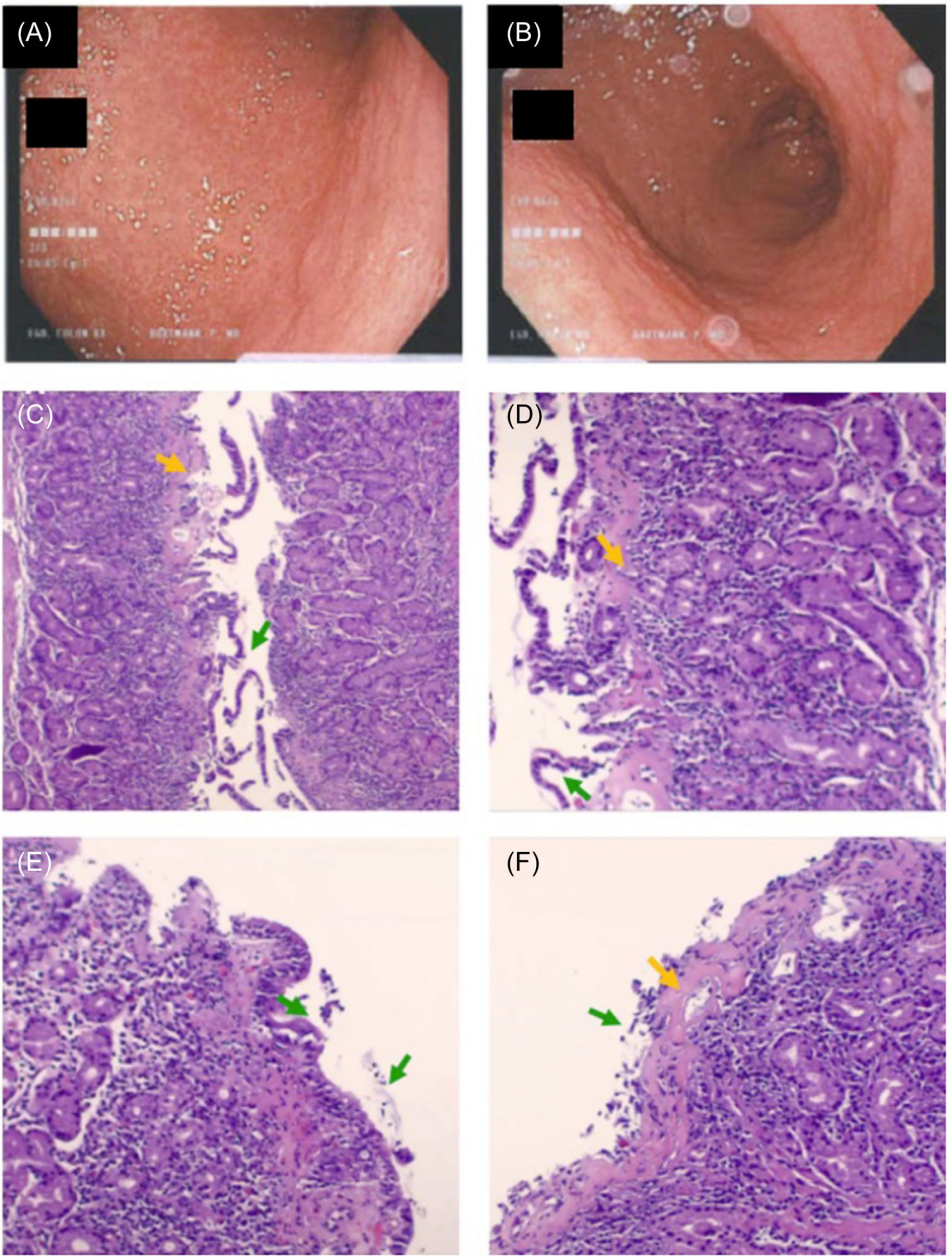
Macroscopic gastric antral mucosa and histological findings of collagenous gastritis. (A, B) Gastroscopy: Diffusely nodular antral gastric mucosa. (C, D) Histopathology of the gastric body with variably thickened subepithelial collagen. (E, F) Histopathology of the gastric antral mucosa with markedly thickened. Orange arrows: Subepithelial collagen. Green arrows: Surface epithelium with denudation and detachment.

An EGD and ileocolonoscopy revealed significant antral nodularity (Figure 1A,B) and one small antral polyp removed by forceps but otherwise macroscopically normal. Microscopically, 12 colonic (two each from the cecum, ascending, transverse, descending, sigmoid, and rectum), eight duodenal, three terminal ileum, six gastric (three each from antrum and body), and six esophageal biopsies were evaluated. The gastric antral and body mucosa showed a thickened subepithelial collagen band with increased intraepithelial lymphocytes and surface epithelial damage with denudation, detachment, and atrophy. The lamina propria revealed prominent chronic inflammation of abundant lymphocytes, plasma cells, and many eosinophils (Figure 1C–F). No iron deposits were identified and all remaining biopsies were microscopically normal. Gram stain and culture of the gastric antral biopsy were negative for *H. pylori*. Magnetic resonance enterography (MRE) was normal.

On follow‐up, our patient reported a decreased frequency of abdominal pain from daily to every other day without intervention. Her home iron supplementation was discontinued. The plan at the submission of this report was to treat with budesonide and fish oil and trend fecal calprotectin with possible further investigation using video capsule endoscopy.

## 3 DISCUSSION

Our case of collagenous gastritis, like other pediatric cases, presented with chronic abdominal pain and was limited macroscopically and histologically to the gastric mucosa.^2^ Our patient had a history of chronic iron deficiency anemia, a common presentation of pediatric collagenous gastritis. However, iron supplementation largely improved her anemia despite developing menorrhagia. Additionally, our patient reported improvement in symptoms without intervention. No reports to our knowledge describe the longitudinal course of collagenous gastritis without intervention. Like other chronic disorders, we suspect a waxing and waning course. In adults, diarrhea is the most common presenting symptom, often linked to concurrent celiac disease and microscopic colitis.^1^ Also, autoimmune and lymphocytic gastritis and duodenal intraepithelial lymphocytosis frequently co‐exist with collagenous gastritis, confounding the diagnosis.^1^ In our case, these associated conditions were not identified.

To our knowledge, this is the first description of collagenous gastritis associated with elevated fecal calprotectin in the absence of significant lower gastrointestinal symptoms and normal colonic biopsies. Only three cases of collagenous gastritis with elevated fecal calprotectin currently exist in the literature. Each presented with significant lower gastrointestinal symptoms reflective of intestinal inflammation: One male aged 15 years with fecal calprotectin of 223 mcg/g with hematochezia^11^ and two females aged 16 and 3 years with fecal calprotectin of 218 mcg/g and 329 mcg/g, respectively, with nonbloody diarrhea.^9^, ^10^ Collagenous *colitis*, a known cause of elevated fecal calprotectin, was concomitantly identified in both female cases. No other cause was identified in the male case, but hematochezia suggests lower gastrointestinal involvement. In our patient, sharp lower abdominal pain was reported, a symptom of colonic inflammation. However, our patient had no diarrhea or hematochezia, and colonic biopsies were normal. Of note, in a review of 15 pediatric collagenous gastritis cases, 67% had elevated *serum* calprotectin, but no data was reported for *fecal* calprotectin.^2^

Fecal calprotectin has been a valuable tool for assessing IBD.^3^ In a review of six adult studies (*n* = 670), the sensitivity and specificity of fecal calprotectin for adult IBD confirmed by endoscopy were 0.93 (95% confidence interval [CI]: 0.85–0.97) and 0.96 (95% CI: 0.79–0.99), respectively. On review of seven pediatric studies (*n* = 371), sensitivity was 0.92 (0.84–0.96), but specificity was 0.76 (0.62–0.86). These findings support the established evidence that non‐IBD causes can elevate fecal calprotectin. These predictably include gastrointestinal infections and other intestinal disorders characterized by inflammation with neutrophilic infiltration.^6^ Yet, it is unclear why fecal calprotectin is elevated in lymphocytic or eosinophilic gastrointestinal conditions and if other sources of calprotectin, such as endothelial cell damage, contribute to this elevation. Our patient's history, laboratory, endoscopy, and imaging ruled out other known causes of elevated fecal calprotectin.

**Figure 2:**
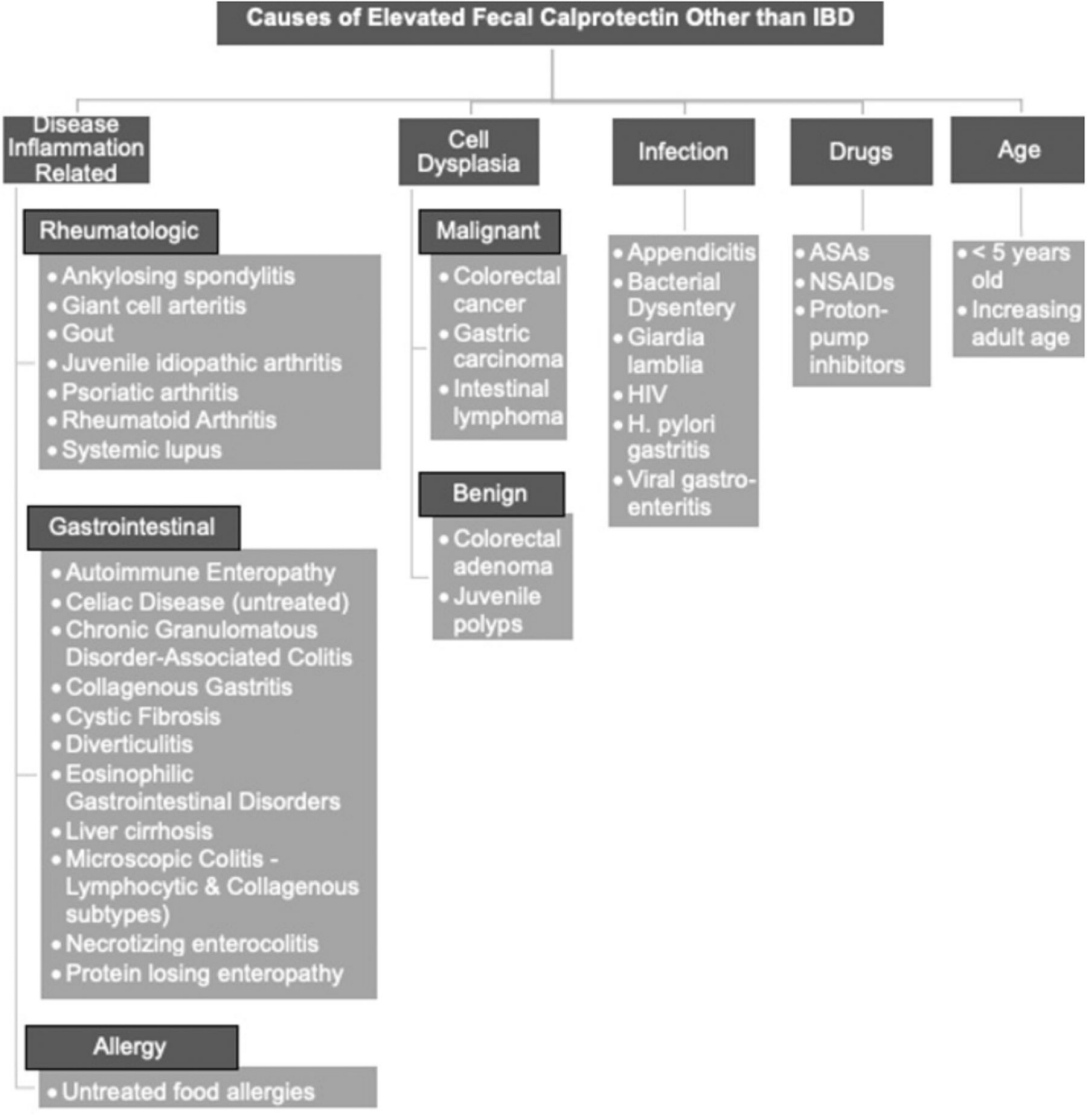
Causes of elevated fecal calprotectin (>50 mcg/g) other than IBD. ASA, acetylsalicylic acid; *H. pylori, Helicobacter pylori*; HIV, human immunodeficiency virus; IBD, inflammatory bowel disease; NSAID, nonsteroidal anti‐inflammatory drug.

In conclusion, elevated fecal calprotectin can be due to collagenous gastritis without intestinal involvement and should be considered in the differential diagnosis (Figure 2).

## CONFLICT OF INTEREST STATEMENT

The authors declare no conflict of interest.

## References

[1] Genta RM , Turner KO , Morgan CJ , Sonnenberg A . Collagenous gastritis: epidemiology and clinical associations. Dig Liver Dis. 2021;53(9):1136‐1140. 33824091 10.1016/j.dld.2021.03.010

[2] Käppi T , Wanders A , Wolving M , et al. Collagenous gastritis in children: incidence, disease course, and associations with autoimmunity and inflammatory markers. Clin Transl Gastroenterol. 2020;11(8):e00219. 32955189 10.14309/ctg.0000000000000219 PMC7431242

[3] D'Angelo F , Felley C , Frossard JL . Calprotectin in daily practice: where do we stand in 2017? Digestion. 2017;95(4):293‐301. 28511188 10.1159/000476062

[4] Klingberg E , Carlsten H , Hilme E , Hedberg M , Forsblad‐d'Elia H . Calprotectin in ankylosing spondylitis—frequently elevated in feces, but normal in serum. Scand J Gastroenterol. 2012;47(4):435‐444. 22229862 10.3109/00365521.2011.648953

[5] Lundgren D , Eklöf V , Palmqvist R , Hultdin J , Karling P . Proton pump inhibitor use is associated with elevated faecal calprotectin levels. A cross‐sectional study on subjects referred for colonoscopy. Scand J Gastroenterol. 2019;54(2):152‐157. 30676120 10.1080/00365521.2019.1566493

[6] van Rheenen PF , Van de Vijver E , Fidler V . Faecal calprotectin for screening of patients with suspected inflammatory bowel disease: diagnostic meta‐analysis. BMJ. 2010;341:c3369. 20634346 10.1136/bmj.c3369 PMC2904879

[7] Yoo IH , Cho JM , Joo JY , Yang HR . Fecal calprotectin as a useful non‐invasive screening marker for eosinophilic gastrointestinal disorder in Korean children. J Korean Med Sci. 2020;35(17):e120. 32356420 10.3346/jkms.2020.35.e120 PMC7200180

[8] Vaos G , Kostakis ID , Zavras N , Chatzemichael A . The role of calprotectin in pediatric disease. BioMed Res Int. 2013;2013:1‐8. 10.1155/2013/542363 PMC3794633 24175291

[9] Remien KA , Mancuso M , Watson K . Pediatric collagenous gastroenteritis and colitis presenting as protein‐losing enteropathy. ACG Case Rep J. 2023;10(4):e01028. 37057196 10.14309/crj.0000000000001028 PMC10090787

[10] Mishra A , Chung J , Mani H , Serrano M . Response to mesalamine therapy in pediatric collagenous gastritis and colitis: a case report and review. Glob Pediatr Health. 2022;9:2333794X221094262. 10.1177/2333794X221094262 PMC9021462 35465197

[11] De Ronde O , Delos M , Jespers S , Gillain C , De Ronde T . Collagenous gastritis: about two paediatric cases and literature review. Acta Gastro‐Enterol Belg. 2020;83(1):41‐45. 32233270

